# Effects of Physical Activity on Motor Skills and Cognitive Development in Early Childhood: A Systematic Review

**DOI:** 10.1155/2017/2760716

**Published:** 2017-12-13

**Authors:** Nan Zeng, Mohammad Ayyub, Haichun Sun, Xu Wen, Ping Xiang, Zan Gao

**Affiliations:** ^1^School of Kinesiology, University of Minnesota-Twin Cities, 1900 University Ave. SE, Minneapolis, MN 55455, USA; ^2^Department of Physical Education, Qujing Normal University, Sanjiang Road, Qujing, Yunnan 655011, China; ^3^College of Biological Sciences, University of Minnesota-Twin Cities, Minneapolis, MN, USA; ^4^College of Education, University of South Florida, 4202 E. Fowler Avenue, EDU105, Tampa, FL 33620-5650, USA; ^5^Department of Physical Education, College of Education, Zhejiang University, 148 Tianmushan Road, Hangzhou 310028, China; ^6^College of Education and Human Development, Texas A&M University, Harrington Education Center Office Tower, 4222 TAMU, 540 Ross Street, College Station, TX 77843, USA

## Abstract

**Objective:**

This study synthesized literature concerning casual evidence of effects of various physical activity programs on motor skills and cognitive development in typically developed preschool children.

**Methods:**

Electronic databases were searched through July 2017. Peer-reviewed randomized controlled trials (RCTs) examining the effectiveness of physical activity on motor skills and cognitive development in healthy young children (4–6 years) were screened.

**Results:**

A total of 15 RCTs were included. Of the 10 studies assessing the effects of physical activity on motor skills, eight (80%) reported significant improvements in motor performance and one observed mixed findings, but one failed to promote any beneficial outcomes. Of the five studies investigating the influence of physical activity on cognitive development, four (80%) showed significant and positive changes in language learning, academic achievement, attention, and working memory. Notably, one indicated no significant improvements were observed after the intervention.

**Conclusions:**

Findings support causal evidence of effects of physical activity on both motor skills and cognitive development in preschool children. Given the shortage of available studies, future research with large representative samples is warranted to explore the relationships between physical activity and cognitive domains as well as strengthen and confirm the dose-response evidence in early childhood.

## 1. Introduction

Physical activity is fundamental to the early development of each child and affects many aspects of a child's health [1]. Contemporary health organizations propose that higher levels of physical activity in school-aged children are associated with important short- and long-term health benefits in physical, emotional, social, and cognitive domains across the life span [2–4]. As such, it is vital to integrate physical activity into the lives of children and set the foundation in facilitating and maintaining a healthy, active lifestyle throughout adulthood [5]. It has been reported that more than 41 million young children under the age of 5 were overweight or obese in 2014, worldwide [6]. The health implications of physical activity during early childhood cannot be disregarded; therefore, it is warranted to investigate the relationships between physical activity and health outcomes and cognition in early ages.

Although early childhood represents a critical period to promote physical activity, the long-term health benefits of being physically active from early ages have yet to be confirmed [7]. It is suggested that promoting physical activity in early childhood may help develop motor skills [8]. This postulation is echoed by evidence showing a reciprocal relationship, albeit cross-sectionally, between physical activity and motor development [9–12]. In fact, motor skills in young children are considered to be linked with various health outcomes such as adiposity [13], self-esteem [14], cardiorespiratory fitness [15], and cognition [16], among others. Hence, developing and implementing effective interventions to improve young children's motor skills have become a priority. As studies examining the effects of physical activity on motor skills continue to increase in frequency, a more recent and thorough review is needed. Although a review study on the topic is available from 2009 [17], the authors failed to include only randomized controlled trials (RCTs), indicating cause-effect relationships cannot be inferred. In addition, the article defined preschool-aged children as aged under 5 years old, which is quite different from national or international interpretation. Therefore, the effectiveness of physical activity interventions on motor skills in this population is still unclear.

Today, advances in neuroscience have generated substantial progress in connecting physical activity to brain structure and cognitive development [18]. It is hypothesized that physical activity has a positive effect on cognitive functions, which is partly due to the physiological changes in the body. For example, increased levels of brain-derived neurotrophic factor (BDNF) can facilitate learning and maintain cognitive functions by improving synaptic plasticity and serving as a neuroprotective agent, which leads to improved neuroelectric activity and increased brain circulation [19]. It is also suggested that one's motor skills may influence cognitive development given that motor and cognitive skills have several common underlying processes, including sequencing, monitoring, and planning [20]. In addition, both motor and cognitive skills may have a similar developmental timetable with accelerated development during childhood [21]. In fact, the literature consistently reports that increased physical activity time in school has no detrimental effect on academic performance and may even enhance academic attainment, executive functions, and on-task behaviors in children and adolescents [19, 22–25]. In addition, emerging evidence suggests that active children tend to have better health and cognitive outcomes when compared to their less active peers [7]. While interest in the relationship between exercise and cognitive functioning has grown over the past decade, the literature concerning the benefits of physical activity on cognition has been addressed in research with older children or adults for the most part. Regrettably, to date, there has been no known comprehensive review specifically examining the effectiveness of physical activity on cognitive outcomes in early childhood.

Early childhood is the most critical and rapid period of complete and healthy motor and cognitive development in human life [26]; increased physical activity may provide motor and cognitive benefits across childhood and adolescence [17, 27]. Therefore, gaining a better understanding of physical activity's potential in improving motor skills and cognition in young children is critical and can inform pediatricians and other health professionals regarding its efficacy as an intervention strategy. There is an urgent need to synthesize RCT studies to definitively establish the presence of effects of physical activity on motor skills and cognitions as well as identify the dose-response relationships for the population of preschool children. Therefore, the purpose of this paper was to systematically evaluate the available evidence examining the effects of physical activity on motor skills and cognitive development in healthy preschool children. Specifically, this systematic review aims to identify, synthesize, and interpret the best available evidence for minimal and optimal amounts of physical activity needed to promote motor skills and cognitive development among children aged 4–6 years. Further, this review attempts to help inform scholars and health professionals concerning the benefits of regular physical activity participation and the development of evidence-based physical activity guidelines for this age group.

## 2. Materials and Methods

The Preferred Reporting Items for Systematic Review and Meta-Analysis Protocols (PRISMA-P) 2015 statement was consulted and provided the structure for this review [28].

### 2.1. Operational Definition

For the purposes of this review, the terms to be used throughout the paper are defined as follows:

Physical activity: any bodily movement produced by skeletal muscles that requires energy expenditure [3], including exercise, active games, and sports programs.

Motor skills: learned sequences of movements that are combined to produce a smooth, efficient action in order to master a particular task [29]. Different categories of motor skills are distinguished in the current review, including fine and gross motor skills, locomotor and object control skills, and body coordination. Notably, the categories are not exclusive, and as such, motor skills from one category may contain elements of other categories [16].

Cognition: the set of mental processes of acquiring knowledge and understanding that contribute to perception, memory, intellect, and action [18]. Different aspects of cognitive functioning were included in this review, such as academic achievement, executive function, learning, language, concentration/attention, memory, and intelligence quotient (IQ).

Preschool children: according to Kail (2011) [30], preschoolers are defined as between 4 and 6 years of age.

### 2.2. Information Sources and Search Strategies

The electronic databases used for the literature search included Academic Search Complete, Communication and Mass Media Complete, Education Resources Information Center (ERIC), Google Scholar, Medline, PsycInfo, PubMed, Scopus, SportDiscus, and Web of Science. The literature search was conducted by the coauthors as a collaborative effort of the research team. Search terms were discussed among the research team and used in combination: (“physical activity” OR “physical education” OR “exercise” OR “sports program”) AND (“motor skill” OR “motor skill competency” OR “motor performance” OR “motor function” OR “motor abilities” OR “motor development” OR “motor coordination” OR “fine motor skills” OR “gross motor skills” OR “locomotor skills” OR “object control skills”) AND (“cognition” OR “cognitive performance” OR “cognitive functions” OR “cognitive abilities” OR “academic achievement” OR “executive function” OR “learning” OR “language” OR “attention” OR “on-task behavior” OR “memory” OR “intelligence” OR “IQ”).

### 2.3. Eligibility Criteria

The following inclusion criteria were used for each study: (1) published in English between January 2000 and July 2017 as peer-reviewed empirical research; (2) sample which was comprised of healthy preschool children (mean age between 4 and 6 years) without chronic diseases and/or physical and mental impairments (e.g., motor disability, autism spectrum disorders, and brain dysfunction); (3) used quantitative measures in the assessment of motor skills and cognitive outcomes; (4) study design which was RCT that assessed the effects of a physical activity or exercise-based intervention. Other study designs, such as cohort and observational studies, were retrieved but excluded in the analysis.

### 2.4. Data Extraction

Three reviewers (NZ, MA, and ZG) independently screened the articles by assessing the article titles. If the reviewers were unable to determine the relevance of an article to the topic, then the abstract was evaluated. Data extraction was completed by one reviewer (MA) and checked by another (NZ) for accuracy. A list of published articles on the topic was then created in a Microsoft Excel spreadsheet. The following information was extracted: (1) year of publication and country of origin; (2) methodological details (e.g., study design, experimental context, sample characteristics, study duration, type of physical activity, outcome measures, and instruments); and (3) key findings with respect to the effectiveness and potential of physical activity on motor and cognitive development (e.g., improved motor performance and reported changes in executive function and on-task behavior). Finally, relevant studies were further identified through cross-referencing the bibliographies of selected articles. Notably, reviewers were not blinded to the authors or journals, and no attempts were made to contact study investigators or correspondents to acquire any information missing from the included articles.

### 2.5. Risk of Bias in Individual Studies

To assess the risk of bias in each study, two reviewers (MA, NZ) independently rated each study on an 8-item quality assessment tool (see Table 1) used in previous literature [31–33]. Each item within each study was rated as “positive” (when the item was explicitly described and present) and “negative” (when the item was inadequately described or absent). Two reviewers (MA, NZ) separately scored each study to ensure reliable scoring of the quality assessment. Unresolved differences were evaluated by a third reviewer (ZG) when disagreements occurred between the two reviewers. Finally, the final score for each study was calculated by summing up the all “positive” rates. A study was considered high-quality study design when scored above the median score following the scoring of all studies.

## 3. Results

### 3.1. Study Selection

**Figure 1:**
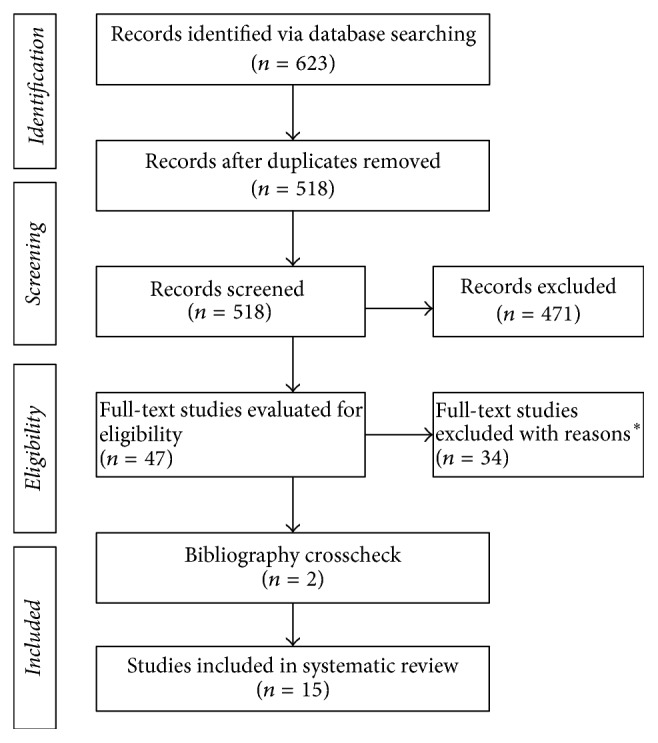
PRISMA flow diagram of studies through the review process. ^*∗*^Reasons for study exclusion included ineligible age, special populations, no measures of motor skills and cognitive development, and non-English language articles. Many studies were excluded for multiple reasons.

A total of 623 articles were identified through a search of the databases. After removing duplicates, titles and abstracts of the remaining articles were screened and further identified as potentially meeting the inclusion criteria. An additional 2 studies were located through the search of reference lists. Following a thorough assessment of the full-text articles, 15 studies fully met the inclusion criteria and were included in this review (see Figure 1). Reasons for excluding articles included ineligible age, special populations, no measures of motor skills and cognitive development, and non-English language articles. Notably, a high interrater agreement (14 out of 15, 93%) of the articles included was obtained between the authors.

### 3.2. Study Characteristics

The characteristics of the included studies are shown in Table 2. Among the 15 RCTs, 10 examined the effects of physical activity on motor skills [34–43] and five assessed the impact of physical activity on cognitive development [27, 44–47]. The studies were conducted in different countries: 5 in Australia [37, 38, 43, 45, 46], 4 in the United States [35, 41, 42, 44], 2 in Switzerland [36, 47], 2 in the United Kingdom [27, 40], 1 in Canada [34], and 1 in Finland [39]. Among these studies, 10 were conducted in childcare center [34–38, 40, 41, 43, 45, 46], 3 were conducted in school settings [27, 44, 47], 1 was conducted at home [39], and 1 was conducted in a laboratory setting [42]. Notably, most of the studies were published after 2010, except for three studies that were published in 2006 [40], 2008 [44], and 2009 [41], indicating research concerning physical activity interventions on motor skills and cognitive development in preschool children is a young, yet expanding, scientific field.

In addition, a relatively large variability in sample size and intervention was observed across studies, with the sample varying from 40 to 625 and intervention length ranging from 4 weeks to 12 months. The exposure in the majority of studies was a physical activity/exercise program or class, while the control group or condition was either usual care or regular school curriculum. The measurement tools used for motor skill and cognitive development outcomes varied across studies but were typically assessments directly completed by children or direct observations made by trained research assistants. Fine and gross motor skills, locomotor and object control skills, executive function, attention, and memory were the most commonly assessed measures of motor performance and cognitive outcomes. Given the heterogeneity of exposures and outcomes, a meta-analysis was unattainable.

### 3.3. Quality and Risk of Bias Assessment

In this review, all included studies were activity-based interventions. Following the ratings of the 8-item quality assessment tool, the design quality and risk of bias for each study were rated from 5 to 8 (see Table 1). Specifically, 2 studies received an overall rating of strong quality/low risk of bias (a study was considered of high quality when scored above the median score of 7 following the scoring of all studies), 7 studies received an overall rating of moderate quality/medium risk of bias, and 6 studies received an overall rating of weak quality/high risk of bias. Notably, all studies succeeded in retaining at least 70% of the participants. The most common issues with the study quality and risk of bias were related to follow-up measurements, power calculations for appropriate sample sizes, and missing data interpretation.

### 3.4. Measurement Protocol

Various types of instrument were used to measure motor skills and cognitive functioning. Specifically, the most common used instrument in assessing children's motor skills was Test of Gross Motor Development-Second Edition (TGMD-2), followed by Peabody Developmental Motor Scales-Second Edition (PDMS-2), Zurich Neuromotor Assessment (ZNA), Körperkoordinationstest für Kinder (KTK), and the Gross Motor Function Measure (GMFM). In addition, cognitive abilities such as attention, memory, language, and academic achievement were evaluated via The Woodcock-Johnson III Tests of Achievement NU (WJ-III ACH), Cambridge Neuropsychological Test Battery (CANTAB), Attention Network Test (ANT), Cognitive Assessment System (CAS) and Connor's Parent Rating Scale (CPRS), Free-Recall and Cued Recall Tests, Konzentrations-Handlungsverfahren für Vorschulkinder (KHV-VK), and the Intelligence and Development Scales (IDS). Notably, measurement tools used for motor skills and cognitive functioning varied across studies. Typically, assessments were directly completed by children or through direct observations made by trained research assistants. Although different instruments were used across various studies, validities of these assessments have been proven when being applied to preschool children in school setting (Table 1).

### 3.5. The Effectiveness of Physical Activity on Motor Skills

Of 10 studies examining the effects of physical activity on preschool children's motor skill outcomes, eight (80%) reported significant improvements in motor development (e.g., fundamental motor skills and motor abilities) following activity-based interventions [34, 35, 37–41, 43]. Notably, one study [42] had mixed findings, observing remarkable enhancements on several variables (i.e., single leg stance test, right grip strength, and left grip strength), with no significant effects found for other outcomes after a Nintendo Wii Sports-based treatments (twice a week × 30 minutes per session for 10 weeks), including gait speed, timed up and go test, five-times-sit-to-stand test, timed up and down stairs test, 2-minute walk test, and gross motor skills assessed by the Gross Motor Function Measure (GMFM). Although significant changes in other outcome measures were not seen between the study groups, there were trends towards greater improvements in the intervention group compared to the control group [42]. It is also worth noting that not all included studies support the effectiveness of physical activity on motor skill development. A governmentally led physical activity program failed to promote any beneficial motor performance outcomes (i.e., climbing up and down the stairs; running; balancing; getting up; and landing after jumping) [36]. The researchers of this particular study highlighted the complexity of implementing physical activity interventions outside of a study setting and urge future similar studies to improve on existing programs [36].

### 3.6. The Effectiveness of Physical Activity on Cognitive Development

Five studies investigated the effects of physical activity on cognitive development in preschool children. Measurements of cognition considered a wide range of cognitive outcomes, including language, academic achievement, attention, working memory, and executive functioning. Amidst these studies, four demonstrated positive effectiveness of activity-based interventions on cognitive functioning while one failed to find significant improvements following a multidimensional lifestyle intervention. Specifically, one study employing a “Tools of the Mind” curriculum guided by the Social Cognitive Theory reported that the experimental group with a strong emphasis on play was found to increase executive functioning, social behavior, language, academic success, and literacy growth compared with control group that used the general education curriculum [44]. In addition, a school-based intervention suggested that children who participated in aerobically intense physical education had significant increases in aspects of cognition and executive functioning when compared to their peers exposed to standard physical education, indicating that the greater degree of neural plasticity of young children may have the most to gain from increased physical activity [27]. Similarly, two studies found that cognitive outcomes were highest in the integrated condition (involving task-relevant physical activities) and higher in the nonintegrated condition (involving task-irrelevant physical activities) than in the control condition (involving the predominantly conventional sedentary style of teaching) [45, 46]. Although a majority of the included studies (80%) support the claim that physical activity promotes cognition in preschool children, one study failed to observe significant changes in young children's attention and spatial working memory after a 10-month multidimensional lifestyle intervention [47]. Notably, cognitive functioning in this study was assessed as a secondary outcome.

## 4. Discussion

The purpose of the current study was to comprehensively evaluate all published RCTs regarding the effects of physical activity on motor skills and cognitive development in apparently healthy preschool children, as well as to provide a synthesis of the current evidence regarding cause and effect relationships. Fifteen studies were included for the final analysis. Findings revealed that increased physical activity had significant beneficial effects on 80% of studies assessing motor skills and cognitive development. Notably, no study found that increased or higher duration/frequency of physical activity had significant detrimental effects on young children's motor skills and cognitive development. Overall, the present systematic review confirms the effectiveness of physical activity; however, the findings were based on a small number of included studies. More studies with larger sample sizes, therefore, are warranted.

Early childhood is considered a critical time period for establishing healthy behaviors such as physical activity [48]. Physical activity programs provide young children with the milieu for motor skill development, with motor skills being the foundation for physical activity during early years and subsequent years [38]. Young children today are showing insufficient proficiency in their motor skills [49]. Indeed, early childhood settings play a significant role in the promotion of physical activity participation and motor skill development since these settings generally have the resources to implement physical activity and motor skill programs [48, 50]. Therefore, interventions to improve young children's motor skills and physical activity have been a priority. Notably, one previous systematic review has examined the effects of activity-based interventions on young children' motor development [17], highlighting the fact that nearly 60% of the included studies (*N* = 17) reported statistically significant improvements at follow-up. However, the majority of included studies (*n* = 12) in that review were quasi-experimental designs. As such, causality with regard to physical activity and motor skills in preschool children is controversial. In addition, of the five included RCTs, two were unpublished doctoral dissertations, and two were published in 1990 and 1996, respectively. As the review was published in 2009 and this area has since received increasing research interest, a more recent and thorough review study is warranted.

The current review included 10 RCTs on the topic of physical activity and motor skills in preschool children. Relative to the question of whether physical activity is causally linked to motor skills, most of the studies (*n* = 8, 80%) have clearly interpreted positive effects of physical activity on motor skills [34, 35, 37–41, 43]. However, it is worth noting that not all included RCTs support the positive effectiveness of physical activity on motor skill development. For example, one study observed mix findings of significant improvements on single leg stance test and grip strength test while no beneficial effects were found for other motor performance tests following a Wii Sports treatment [42]. Although no significant changes were detected in other outcome measures, trends towards greater enhancements in the experimental group emerged [42]. In addition, a 9-month governmentally led physical activity program did not result in increased motor skill performance [36]. One possible explanation for these different findings would be that the intervention did not provide participants with a sufficient physical activity dose. It is also possible that the modest sample size may have contributed to the decrease in the significance of these measures. Of the eight efficacious RCTs, intervention length ranged from 9 weeks to one year with more than half of the interventions being longer than 5 months. Notably, most treatments used supervised physical activity programs of approximately 30 minutes for 3 times per week at a childcare or home-based setting. In fact, effectiveness of physical activity programs may be affected by many factors during implementation and assessment stages. Given the fact that each RCT was uniquely distinct in intervention delivers, content, instructional methods, and measurements and that no precise mandatory demands were made by most studies with regard to the physical activity dose, it is difficult to identify specific intervention components that contributed to effectiveness. Nonetheless, strong evidence from these 8 efficacious RCTs suggests that a greater amount of physical activity led by teachers or parents would be necessary to achieve more beneficial effects on young children's motor skill development in ordinary, daily circumstances. This allows for conclusions to be drawn concerning cause and effect relationships between physical activity and motor skills in preschool children. Overall, evidence regarding the effectiveness of physical activity interventions on motor skill development is strong. Nevertheless, identifying the dose of physical activity intervention that aims to improve preschool children's motor skills should be the focus of future research.

Early childhood is considered one of the most critical and intensive periods of brain development throughout the human lifespan [50], and habitual physical activity is a key determinant of cognition during childhood [8]. Today, a growing body of literature suggests that physical activity has beneficial effects on cognitive development, such as attention, working memory, classroom behavior, and academic achievement among children and youth [51–54]. In addition, it is believed that motor skills and cognitive development are closely related as both motor and cognitive skills have several common underlying processes including sequencing, monitoring, and planning [20]. Recent literature has reviewed relationships between motor skills and cognition in 4–16 year children and suggested that weak-to-strong relations exist between two variables [16]. The authors concluded that complex motor intervention programs may be necessary to stimulate motor skills and higher order cognitive development in children. Regrettably, there is no literature available investigating the effects of motor skill intervention on cognitive development in young children. In contrast, the use of a physical activity intervention has generated substantial public interest for young children's cognitive development. One recent study has reviewed the relationships between physical activity and cognitive development during early childhood (birth to 5 years) [53]. The authors concluded that physical activity may have beneficial effects on cognitive development during early childhood. However, six of the seven included studies were rated weak quality with a high risk of bias in the review, and no RCT studies were included. That is, the effectiveness of physical activity on preschool children's cognitive development is still unknown.

Five RCTs examining cause and effect relationships of physical activity and cognitive development were included in the current review. In general, evidence of the effectiveness of physical activity on preschool children's cognitive outcomes is favorable, with four studies (80%) [27, 44–46] indicating positive effects while one study reported no effect [47]. The finding of the present review is in line with previous reviews indicating a positive association in the same direction among children, youth, and adults [55, 56]. Although research evidence in other age groups supports the importance of physical activity for cognitive health, findings in older children and adults cannot be generalized to preschool children given the unique developmental differences across age groups [53]. Our study, therefore, is worthwhile in presenting solid evidence to the field. Of the four efficacious RCTs, one observed significant changes in language and academic achievement after 8-month treatment [44], one found improvements in cognitive functions test after 10 weeks [27], and two showed increased learning and working memory following a 4-week intervention [45, 46]. Three studies involved task-relevant physical activities [44–46] while one used aerobically intense physical education (2 hours/week × 10 weeks) [44]. Nevertheless, one study employing a multidimensional lifestyle intervention (i.e., physical activity, nutrition lesson, media use, and sleep management) failed to improve children's attention and spatial working memory following a 10-month treatment [47]. This could be attributed to the physical activity program in this study being designed as playful and organized into different themes, despite the fact that children participated in four 45 minute sessions of physical activity a week. That said, task-irrelevant physical activity may not be beneficial for improving children's attention and spatial working memory. Overall, there is small but strong evidence supporting the causal relationship between physical activity and cognitive functioning in healthy preschool children, with high intensity and task-relevant physical activity being more beneficial for cognitive development in this age group. Given the limited number of studies included in the review, more RCTs are warranted to strengthen the evidence base and confirm the importance of dose (i.e., duration, intensity, frequency, and type) of physical activity for optimal cognitive development in preschool children.

While this systematic review offers a timely and comprehensive investigation into the effect of physical activity on preschool children's motor skills and cognitive development, there are some limitations worth noting when interpreting the findings. First, the current review only included peer-reviewed full-text and English language publications, despite the fact that other unpublished and non-English research may be available on the topic. Second, as most included studies were from Western countries, unrepresentative samples may limit the ability to generalize findings to other regions and populations, such as developing countries and other ethnicities/races. Third, varied measurement protocols may lower interunit variability due to different assessments employed to preschool children among selected studies. Fourth, it is worth noting that the moderating effect may change the strength of an effect or relationship between the independent variable and the outcome variable. For example, PA intervention type might be a moderator in that school-based physical education program may be more effective in promoting motor skills than home-based health education program. Last, given a small number of empirical studies, conclusive statements concerning the effectiveness of physical activity on preschool children's motor skills and cognitive development must be interpreted with caution and therefore state the need for greater study.

## 5. Conclusion

Today, young children are sedentary for significant portion of the day [56]. Since early childhood is regarded as an important period of motor and cognitive development, understanding the effects of physical activity on motor skills and cognitive development in preschool children has major public health implications. This systematic review synthesizes the high-quality experimental evidence available regarding the effectiveness of physical activity on motor skills and cognitive development in 4–6-year old, typically developing children. Findings favor causal evidence of relations between physical activity with both motor skills and cognitive development in preschool children, with increased physical activity having significant beneficial effects on motor skills and cognitive functioning. Given the small number of studies available in the literature, future research with large representative samples is needed to explore other cognitive domains (e.g., executive function and intelligence) and to strengthen and confirm the dose-response evidence.

## Figures and Tables

**Table 1:** Design quality analysis.

Articles	Randomization	Control	Pre-post	Retention	Missing data	Power analysis	Validity measure	Follow-up	Score	Effectiveness
Adamo et al. [34]	+	+	+	+	−	−	+	−	5	Yes
Bellows et al. [35]	+	+	+	+	−	−	+	+	6	Yes
Bonvin et al. [36]	+	+	+	+	+	+	+	+	8	NA
Hardy et al. [37]	+	+	+	+	−	+	+	+	7	Yes
Jones et al. [38]	+	+	+	+	−	+	+	−	6	Yes
Laukkanen et al. [39]	+	+	+	+	+	−	+	+	7	Yes
Reilly et al. [40]	+	+	+	+	−	+	+	+	7	Yes
Robinson & Goodway [41]	+	+	+	+	+	−	+	−	6	Yes
Salem et al. [42]	+	+	+	+	+	+	+	−	7	Yes/NA
Zask et al. [43]	+	+	+	+	+	+	+	−	7	Yes
Barnett et al. [44]	+	+	+	+	−	+	+	−	6	Yes
Fisher et al. [27]	+	+	+	+	+	+	+	−	7	Yes
Mavilidi et al. [45]	+	+	+	+	+	−	+	+	7	Yes
Mavilidi et al. [46]	+	+	+	+	+	−	+	−	6	Yes
Puder et al. [47]	+	+	+	+	+	+	+	+	8	NA

*Note*. “+” refers to positive (explicitly described and present in details); “−” refers to negative (inadequately described and absent); “Yes” indicates significant positive effect; NA indicates no significant effect; +/NA represents significant improvements which were found on several measures while no significant effects were observed on other measures; median score = 7.

**Table 2:** Descriptive characteristics of included RCTs.

Reference	Sample	Testing/setting	Outcomes/instrument	Exposure	Dose	Findings
Adamo et al. [34] 2016, Canada	*N* = 75 (3–5 years); intervention (*n* = 36, M_age_ = 3.4 years, SD = 0.3), control (*n* = 39, M_age_ = 3.4 years, SD = 0.4)	Pre-post; childcare setting	Fundamental movement skills measured via the Test of Gross Motor Development-2nd Edition (TGMD-2)	Intervention group received “The Preschoolers Activity Trial” consisted of the delivery of training workshops to teach the childcare providers how to foster a childcare environment that provides ample opportunities to be physically active throughout the day, while control received standard childcare curriculum during the study period	6 months	The intervention group showed a significantly greater increase in locomotor skills than the control group

Bellows et al. [35] 2013, USA	*N* = 201 (111 boys); intervention (*n* = 98, M_age_ = 53 months, SD = 6.8), control (*n* = 103, M_age_ = 51.5 months, SD = 6.6)	Pre-post; childcare setting	Motor skill performance: gross motor skills measured via the Peabody Developmental Motor Scales- 2nd Edition	Intervention group received “Get Moving with Mighty Moves Program,” while control group received no intervention	The Mighty Moves intervention lasted 18 weeks and was conducted in the classroom 4 days per week for 15–20 min each day, for a total of 72 lessons	The intervention group had significant changes in gross motor skills compared with the control group

Bonvin et al. [36] 2013, Switzerland	*N* = 58 (M_age_ = 3.3 months, SD = 0.6); intervention (*n* = 29), control (*n* = 29)	Pre-post; childcare setting	Motor skills: climbing up and down the stairs; running; balancing; getting up; landing after jumping measured via Zurich Neuromotor Assessment (ZNA)	Intervention group received a governmentally led center based child care physical activity program (real-life), while control group received no intervention	9 months	The intervention group showed no significant increase in motor skills compared to the control group

Hardy et al. [37] 2010, Australia	*N* = 347; intervention (*n* = 213, M_age_ = 4.4 years, SD = 0.5), control (*n* = 134, M_age_ = 4.5 years, SD = 0.3)	Pre-post; childcare setting	Fundamental movement skills (FMS) measured via TMGD-2	Intervention group received “Munch and Move” program which was developed to enhance children's healthy eating, active play, and fundamental movement skills. Control group received health information on unrelated topics (road safety and sun safety)	6 months	Locomotor, object control, and total FMS scores significantly improved in the intervention group compared with the control group

Jones et al. [38] 2011, Australia	*N* = 97 (M_age_ = 4.13 years); intervention (*n* = 52), control (*n* = 45)	Pre-post; childcare setting	Movement skill competence assessed via TGMD-2	Intervention group received structured activities, while control group received usual care activities	20 minutes a lesson × 3 times a week for 20 weeks	The intervention group showed greater improvements in movement skill proficiency compared with the control group

Laukkanen et al. [39] 2015, Finland	*N* = 91 (42 boys; M_age_ = 6.2 years, SD = 1.1); intervention (*n* = 46), control (*n* = 45);	Baseline, 6 and 12 months; home setting	Motor competence: walking backwards, hopping for height, jumping sideways (JS) and moving sideways via Körperkoordinations Test für Kinder (KTK); ball-handing skills via a throwing and catching a ball test (TCB)	Intervention group received family-based physical activity counseling, while control group received no counseling	12 months; parents received a lecture (30 minutes) and face-to- face/phone counseling with goal setting (30–60 minutes) at 2 and 5 months	The intervention group was found to increase motor coordination

Reilly et al. [40] 2006, UK	*N* = 545; intervention (*n* = 268, 128 boys, M_age_ = 4.2 years, SD = 0.3), control (*n* = 277, 145 boys, M_age_ = 4.1 years, SD = 0.3)	Baseline, 6 and 12 months; childcare setting	Motor skills: jumping, balance, skipping, and ball exercises measured via Movement Assessment Battery	Intervention group received enhanced physical activity program plus home-based health education aimed at increasing physical activity through play and reducing sedentary behavior, while control group received usual curriculum	3 × 30 minute sessions a week for 24 weeks	The intervention group had significantly higher performance in movement skills than control group at six-month follow-up

Robinson & Goodway [41] 2009, USA	*N* = 117 (M_age_ = 4.13 years); low-autonomy (LA) (*n* = 38, 20 boys, M_age_ = 46.6 months, SD = 5.9); mastery motivational climate (MMC) (*n* = 39, 19 boys, M_age_ = 47.6 months, SD = 7.5); control (*n* = 40, 24 boys, M_age_ = 48.3 months, SD = 5.0)	Pre-post; childcare setting	Object control skill was measured via TGMD-2	LA and MMC groups received object control skill intervention while control group received typical Head Start curriculum	30 minutes, 2 days per week for 9 weeks	Significant improvements in object control skills were present for both intervention groups while the control group resulted in no changes

Salem et al. [42] 2012, USA	*N* = 40 (22 boys); intervention (*n* = 20, M_age_ = 49.3 months, SD = 1.1), control (*n* = 20, M_age_ = 48 months, SD = 5.8)	Pre-post; laboratory setting	Motor abilities: gait speed, timed up and go test, single leg stance test, five-times-sit-to-stand test, timed up and down stairs test, 2-minute walk test and grip strength. Gross motor skills were measured via the Gross Motor Function Measure (GMFM)	Intervention group received Nintendo Wii Sports and Nintendo Wii Fit, including balance, strength training and aerobics games, while control group received traditional sessions that focused on facilitation of movement transitions, balance, walking, and gross and fine motor control	Two 30-minute weekly individual sessions over a period of 10 weeks	Significant improvements in intervention group were observed in single leg stance test, right grip strength and left grip strength compared with the control group

Zask et al. [43] 2012, Australia	*N* = 31 schools (3–6 years children); intervention (*n* = 18 schools), control (*n* = 13 schools)	Pre-post; childcare setting	Fundamental movement skills measured via TGMD-2	Intervention schools received “The Tooty Fruity Vegie in Preschools program”	Structured twice-weekly FMS development through prescribed games suitable for a wide age range for 10 months	The intervention group significantly improved movement skills compared with the control group

Barnett et al. [44] 2008, USA	*N* = 210 (3-4 years); intervention (*n* = 88), control (*n* = 122)	Pre-post; school setting	Cognitive abilities, language, and academic achievement measured via Woodcock–Johnson Applied Math Problems and Letter–Word Identification Tests, Get Ready to Read, the Wechsler Preschool Primary Scale of Intelligence Animal Pegs subtest, the Peabody Picture Vocabulary Test-III (PPVT-III), Expressive One-Word Picture Vocabulary Test (EOWPVT), and the Oral Language Proficiency Test	Intervention group received The Tools of the Mind curriculum, while control group received an established district-created model	Intervention teachers received 4 full days of curriculum training before classes began. During the school year intervention teachers received 30 min classroom visits once a week to address any difficulties they were having with the curriculum. Intervention from October 2002 to June 2003	The intervention group was found to increase executive functioning, social behavior, language, academic success, and literacy growth compared with the control group

Fisher et al. [27] (2011), UK	*N* = 64 (33 boys; M_age_ = 6.2 years, SD = 0.3); intervention (*n* = 34), control (*n* = 30);	Pre-post; school setting	Cognitive functions: Cambridge Neuropsychological Test Battery (CANTAB), the Attention Network Test (ANT), the Cognitive Assessment System (CAS), and the short form of the Connor's Parent Rating Scale (CPRS)	Intervention group received aerobically intense physical education, while control group received standard PE	2 hours per week × 10 weeks for both groups	The intervention group was found to improve CANTAB Spatial Span, CANTAB Spatial Working Memory Errors, and ANT Accuracy compared with the control group

Mavilidi et al. [45] 2015, Australia	*N* = 111 (64 boys; M_age_ = 4.94 years, SD = 0.56); integrated condition (*n* = 31), nonintegrated condition (*n* = 23), gesturing condition (*n* = 31), conventional condition (*n* = 26)	Week 2, 4, and 10; childcare setting	Memory performance was measured via Free-Recall and Cued Recall Tests	Integrated condition: children enacted the actions indicated by the words to be learned in physical exercises; nonintegrated condition: children performed physical exercises at the same intensity, but unrelated to the learning task; gesturing condition: children enacted the actions indicated by the words to be learned by gesturing while remaining seated; conventional condition: children verbally repeated the words while remaining seated	Participants learned 14 Italian words in a 4-week teaching program	Children in the integrated physical exercise condition achieved the highest learning outcomes

Mavilidi et al. [46] 2017, Australia	*N* = 86 (45 boys, M_age_ = 4.90 years, SD = 0.52); integrated condition (*n* = 30, M_age_ = 4.90 years, SD = 0.52), nonintegrated condition (*n* = 27, M_age_ = 4.96 years, SD = 0.51), control (*n* = 29, M_age_ = 4.80 years, SD = 0.44)	Baseline, week 4, week 6; childcare setting	Learning and memory were measured via Free-Recall and Cued Recall Tests	An integrated physical activity condition including task-relevant physical activities, a nonintegrated physical activity condition involving task-irrelevant physical activities, or a control condition involving the predominantly conventional sedentary style of teaching	Once per week for 4 weeks	Learning outcomes were highest in the integrated condition and higher in the nonintegrated condition than in the control condition

Puder et al. [47] 2011, Switzerland	*N* = 625 (326 boys); intervention (*n* = 167, M_age_ = 5.2 years, SD = 0.6), control (*n* = 159, M_age_ = 5.2 years, SD = 0.6)	Pre-post; school setting	Cognitive abilities: attention and spatial working memory measured via Konzentrations-Handlungsverfahren für Vorschulkinder (KHV-VK) and the Intelligence and Development Scales (IDS)	Intervention group received a multidimensional lifestyle treatment, while control group did not receive any treatment and continued their regular school curriculum	Children participated in a physical activity program consisting of four 45 minute sessions of physical activity a week from August 2008 to June 2009	No significant differences between groups
